# Dietary supplementation of dried plum: a novel strategy to mitigate heat stress in broiler chickens

**DOI:** 10.1186/s40104-021-00571-5

**Published:** 2021-03-30

**Authors:** Sanjeev Wasti, Nirvay Sah, Amit K. Singh, Chin N. Lee, Rajesh Jha, Birendra Mishra

**Affiliations:** Department of Human Nutrition Food and Animal Sciences, University of Hawaii at Manoa, Honolulu, HI 96822 USA

**Keywords:** Dried plum, Gene expression, Heat stress, Microbiota, Mitigation

## Abstract

**Background:**

Heat stress is a significant problem in the poultry industry, causing a severe economic loss due to its detrimental effects on chickens’ health and performance. Dried plum (DP) is a good source of minerals, vitamins, antioxidants, and phenolic compounds. Studies have suggested that DP has several health benefits, such as maintaining the body’s redox system, immune status, and calcium hemostasis. Based on the health benefits of DP, we hypothesized that the dietary supplementation of DP would alleviate the detrimental effects of heat stress on broiler chickens.

**Results:**

To test the hypothesis, day-old broiler chicks (*n* = 72) were randomly allocated to three treatment groups (*n* = 24/group): no heat stress (NHS), heat stress (HS), and heat stress with dried plum (HS + DP), and reared under standard conditions. The inclusion of 2.5% DP in the feed of the HS + DP group was made during the treatment period, while birds in other groups were provided with a standard finisher diet. After 21 days, birds in the HS and HS + DP groups were exposed to cyclic heat stress conditions (33 °C for 8 h during daytime) for 3 weeks, while those in the NHS group were reared under normal conditions (22–24 °C). Weekly body weight and feed intake were recorded to calculate the average daily gain (ADG), average daily feed intake (ADFI), and feed conversion ratio (FCR). Heat stress significantly decreased the final body weight, ADG, ADFI, and increased FCR compared to the NHS group, whereas dietary supplementation of DP significantly improved these growth performance parameters compared to the HS group. Furthermore, supplementation of DP significantly increased the expression of heat shock protein-related genes (*HSF1, HSF3, HSP70,* and *HSP90*), antioxidant-related genes (*SOD1, SOD2, GPX1, GPX3, PRDX1,* and *TXN*), tight junction-related genes (*CLDN1,* and *OCLN*), and immune-related genes *(IL4, MUC2)* in the ileum as compared to the HS group*.* The microbiota analysis showed significant enrichment of Bacillales, Christensenellaceae, Bacillaceae, Peptostreptococcaceae, and *Anaerotruncus* in heat-stressed birds supplemented with DP as compared to the HS group. Further, DP supplementation also significantly increased the concentration of acetate, propionate, and total VFA in the cecal digesta of the HS + DP group as compared to the HS group.

**Conclusion:**

These findings suggest that DP supplementation effectively improved the growth performances and gut health parameters in the heat-stressed birds. Thus, dried plum can be a potential feed supplement to mitigate heat stress in broiler chickens.

**Supplementary Information:**

The online version contains supplementary material available at 10.1186/s40104-021-00571-5.

## Introduction

High environmental temperature is a significant problem in the poultry industry, affecting the health and performance of poultry, which is expected to worsen due to increasing global warming. It is estimated that heat stress results in an annual economic loss of $128 to $165 million to the US poultry industry [1]. Heat stress in poultry results in several physiological changes such as oxidative damage, acid-base imbalance, and suppressed immunocompetence, leading to reduced feed intake, poor feed efficiency, and reduced body weight, poor meat quality, increased susceptibility to diseases, and higher mortality [2]. Poultry meat is the most consumed and widely accepted animal protein worldwide, and its demand is ever increasing to meet the growing population. There has been a huge improvement in chicken genetics over the past decades to meet this demand [3]. These improved strains have higher metabolic rates and production performances [4] and are prone to higher environmental temperatures.

It is practically impossible to avoid heat stress in poultry farms; however, several strategies have been successfully applied to improve the deleterious effects of heat stress in poultry [2]. As environmental heat stress exerts its detrimental effects by inducing oxidative stress, various strategies such as supplementation of vitamins, minerals, antioxidants, and plant extracts have been used to reduce oxidative stress in poultry [5]. Phytochemicals with antioxidant activity have been emerged as a better solution to improve heat stress in poultry [2, 6].

Dried plum (*Prunus domestica *L.) has the highest oxygen radical absorbance capacity (ORAC) score among the 22 most commonly consumed vegetables and fruits [7, 8]. DP also contains a reasonable amount of antioxidants (fat-soluble carotenoids, alpha-tocopherol, etc.), polyphenolic compounds (such as chlorogenic acids, proanthocyanidins, etc.), sorbitol, and fibers [9]. Moreover, DP is a good source of several vitamins (vitamin A, C, K_1_, B_1_, B_2_, and niacin) and minerals (Ca, K, Mg, Se, and Zn) [9] (Table S1). DP, a mixture of such bioactive compounds, is known to minimize oxidative stress in rodents [7]. Besides its antioxidant role, DP has several beneficial effects on gut health, calcium metabolism, and immune function [9, 10]. Several *in vitro* studies have highlighted the effectiveness of DP to prevent free radical damage as well as inflammatory responses [11, 12]. DP also serves as a supplemental feed additive due to its wide availability in different parts of the world at a reasonable price, considering the claimed benefits. However, there is no reported study on the effectiveness of DP in ameliorating heat stress effects in poultry. Based on the noted health benefits of DP, we hypothesized that the dietary supplementation of DP would mitigate the detrimental effects of heat stress on gut health and growth performances in poultry. This study aimed to determine the mitigatory effects of DP on growth performance and the gut health parameters in heat-stressed broiler chickens.

## Material and methods

### Animals and husbandry practices

All the animal experiments were carried out following the approved protocol from the University of Hawaii Institutional Animal Care and Use Committee (IACUC) (Approval No.17-2605). The dose of DP (2.5%) was chosen based on its antioxidant contents and the dose rate used in the human and rodent studies. Day-old Cob-500 unsexed chicks (*n* = 72) were sourced from a local hatchery, weighed individually, winged tagged, and placed equally and randomly in 18 pens (4 birds per pen), making 6 replicates of each treatment (*n* = 24 per treatment). Bodyweight (average weight of 40–41 g) was considered to allocate the chicks in each treatment group. The treatment groups were: 1) no heat stress with basal diet (NHS), 2) heat stress with basal diet (HS), and 3) heat stress with basal diet and dried plum (HS + DP) (Fig. S1). Birds were raised on the floor pen system following the standard broiler rearing guidelines for the first 21 d. After 21 d, birds in the HS and HS + DP groups were exposed to 33–35 °C (during the day- 8:00 to 18:00) and 21–22 °C (during the night) with 50% relative humidity for 3 weeks. Birds in the NHS group were reared in identical rooms as HS, and HS + DP groups at standard room temperature (22–24 °C) with 50% relative humidity. Birds were monitored twice daily (in the morning and the evening) for health conditions. A completely randomized design was used in this study. The pen’s size was 1 m × 0.61 m, and the stocking density was 1500 cm^2^/bird. The lighting regime was 23 h light and 1 h dark period.

### Diet

Birds were provided with ad libitum feed and water. The diets were prepared in two phases: starter (1–21 d) and finisher (22–42 d) to meet the nutrients requirements of broilers [13]. Birds were provided with the standard starter diet for the first 14 d. Afterward, from 14 to 21 d, the inclusion of 2.5% of DP was made in the starter diet in the HS + DP, while the other two groups were provided with the standard starter diet. From 21 to 42 d, NHS and HS birds were provided with the standard finisher diets, and the inclusion of 2.5% DP was made in the standard finisher diet of the HS + DP group (Table 1). The available nutrient profile of the DP [9] is presented in Table S1.

**Table 1:** Ingredients and nutrient composition of the experimental diets

	Starter		Finisher	
Ingredients, %	Standard	With DP	Standard	With DP
Corn	54.86	53.36	63.14	61
SBM	39.5	38.5	29.6	29
Dried plum	0	2.5	0	2.5
Soybean oil	2	2	4.5	4.74
Limestone	1.27	1.27	0.85	0.85
Monocalcium phosphate	0.75	0.75	0.5	0.5
Lysine	0.23	0.23	0.18	0.18
Methionine	0.14	0.14	0.12	0.12
Threonine	0.2	0.2	0.16	0.16
NaCl	0.43	0.43	0.35	0.35
Sodium bicarbonate	0.12	0.12	0.1	0.1
Vitamin + Mineral mix*	0.5	0.5	0.5	0.5
Calculated nutrient contents, %				
MEn, kcal/kg	2909	2903	3203	3207
CP	22.09	21.96	18.07	18.08
Ca	0.75	0.75	0.52	0.52
Total P	0.57	0.56	0.47	0.46
dig P	0.30	0.30	0.23	0.23
Lysine	1.39	1.36	1.10	1.08
Methionine	0.48	0.47	0.41	0.41
Cystine	0.43	0.41	0.38	0.37
Threonine	1.03	1.01	0.85	0.83
Tryptophan	0.33	0.32	0.26	0.25
Methionine + Cysteine	0.91	0.89	0.8	0.78
Arginine	1.61	1.57	1.31	1.28
Valine	1.22	1.19	1.03	1.00
Isoleucine	0.93	0.91	0.76	0.75
Leucine	1.89	1.84	1.63	1.59
Choline, mg/kg	1419	1382	1200	1170
dig Lys	1.25	1.22	0.99	0.97
dig Met	0.45	0.45	0.39	0.38
dig Thr	0.85	0.83	0.69	0.68
NDF	9.13	8.89	8.78	8.53
CF	3.97	4.29	3.46	3.8
Na	0.22	0.22	0.18	0.18
Cl	0.30	0.30	0.25	0.25

*Providing the following (per kg of diet): vitamin A (trans-retinyl acetate), 10,000 IU; vitamin D_3_ (cholecalciferol), 3000 IU; vitamin E (all-rac-tocopherol-acetate), 30 mg; vitamin B_1_, 2 mg; vitamin B_2_, 8 mg; vitamin B_6_, 4 mg; vitamin B_12_ (cyanocobalamin), 0.025 mg; vitamin K_3_ (bisulphatemenadione complex), 3 mg; choline (choline chloride), 250 mg; nicotinic acid, 60 mg; pantothenic acid (*D*-calcium pantothenate), 15 mg; folic acid, 1.5 mg; betaíne anhydrous, 80 mg; *D*-biotin, 0.15 mg; zinc (ZnO), 80 mg; manganese (MnO), 70 mg; iron (FeCO_3_), 60 mg; copper (CuSO_4_·5H_2_O), 8 mg; iodine (KI), 2 mg; selenium (Na_2_SeO_3_), 0.2 mg

### Growth performance

Birds were weighed individually at the start of experiments and were placed in replicate pens. Then, weekly (7, 14, 21, 28, 35, and 42 d) body weight and feed intake per replicate pen were recorded. Based on these data, average daily gain (ADG), average daily feed intake (ADFI), and feed conversion ratio (FCR) were calculated.

### Sample collection

At the end of the animal experiment (42 d), two birds from each pen (12 per treatment) were euthanized using carbon dioxide asphyxiation. The sampling was done after a few hours of heat stress during 8 h high temperature. For the ileal gene expression study, small pieces of the ileum (5 cm proximal to the ileocecal junction) were collected (*n* = 6 per treatment; one from each pen), snap-frozen, and stored at − 80 °C until RNA extraction. For the ileum histomorphology (*n* = 4 per treatment), approximately 1 cm of the ileum sample (6 cm proximal to the ileocecal junction) was excised and flushed with 0.9% normal saline to clear the intestinal digesta. The ileal tissues were sent to the histology core facility at John A. Burns School of Medicine of the University of Hawaii at Manoa for embedding, sectioning, and staining with Hematoxylin and Eosin (H&E). For the microbiota characterization (*n* = 6 per treatment; one from each pen) and VFA analysis (*n* = 12 per treatment; two from each pen), the cecum was excised and wrapped separately in aluminum foil and was snapped frozen at − 80 °C. Tissues were fixed overnight in 10% neutral buffered formalin (NBF, pH 7).

### Quantitative real-time PCR (qPCR)

Total RNAs were isolated from the frozen tissues (50–100 mg) using TRIzol reagent (Invitrogen, Carlsbad, CA, USA) according to the manufacturer’s instructions. The total RNA concentration was measured using NanoDrop One (Thermo Fisher Scientific, Madison, WI, USA). The quality of the RNA was determined by running samples on 2% agarose gel. The RNA samples were stored at − 80 °C until further analysis. The expressions of candidate genes were analyzed using qPCR as previously described [14]. Specific primer pairs for detecting each gene were designed using the NCBI Primer-Blast tool (Table S2). Briefly, complementary DNA (cDNA) was synthesized from 1 μg of total RNA (20 μL reaction of RT mixture) using a High-Capacity cDNA Reverse Transcription Kit (Applied Biosystems, Foster City, CA, USA) and further diluted with nuclease free-water (1:25). The qPCR was performed using PowerUp SYBR Green Master Mix (Applied Biosystems, Foster City, CA, USA) on a StepOne Plus real-time PCR system (Applied Biosystems). The qPCR reaction mixture consisted of 3 μL of cDNA, 5 μL PowerUp SYBR Green Master Mix, and 1 μL of each forward and reverse primers (5 μmol concentration) to make a final reaction mixture of 10 μL. The qPCR reaction was carried out following standard cycling mode. A melting curve was also generated to confirm SYBR Green-based objective amplicon. Further, each primer pair’s specificity was determined by running the qPCR products on 1% gel electrophoresis. The three different housekeeping genes: glyceraldehyde 3-phosphate dehydrogenase (GAPDH), beta-actin (β-actin), and TATA-box binding protein (TBP), were analyzed in triplicate in all treatment groups. β-actin was the most stable housekeeping genes in the ileum, and it was used for the normalization of gene expression. The target genes were analyzed in duplicates, and an average value was taken for each experimental replicate. The expression levels of candidate genes in this study were determined using the cycle threshold (Ct) values following the standard curve method after normalization with β-actin. The fold change for each gene was calculated by the 2^−^^ΔΔCt^ method.

### Ileum histomorphology

Ileal tissues fixed in 10% NBF were first dehydrated with a series of ethanol solutions (70%, 80%, 95%, and 100%) and were finally embedded in paraffin as described previously [15]. The embedded ileal tissue was sectioned at 6 μm thickness and stained with H&E. A total of 6 intact, well-oriented villus-crypt units were selected in triplicate (18 measurements for each sample). Sections were observed under an 8× objective lens, and images were taken using an Olympus microscope (U-TV0.63XC, Tokyo, Japan). Different intestinal morphological parameters such as villus height (VH)- distance from the tip of villus to the crypt, crypt depth (CD)- distance from villus base to the submucosa, and the ratio of villus height to crypt depth (VH/CD) were measured by using Infinity Analyze software (Lumenera Corporation, Ottawa, ON, Canada). The apparent villus surface area (VSA) was calculated using the formula proposed by [16].

$$ \mathrm{VSA}=\left(\mathrm{villus}\ \mathrm{width}\ \mathrm{at}\ 1/{3}^{\mathrm{rd}}+\mathrm{villus}\ \mathrm{width}\ \mathrm{at}\ 2/{3}^{\mathrm{rd}}\ \mathrm{of}\ \mathrm{the}\ \mathrm{villus}\ \mathrm{height}\right)\times {2}^{-1}\times \mathrm{villus}\ \mathrm{height} $$

### Volatile fatty acids (VFA)

VFA was analyzed as previously described [17]. Briefly, 200 mg of the cecal content was weighed in the Eppendorf tube. Then, 100 μL of an internal standard, Trimethyl acetate (TMA), and 200 μL of 25% metaphosphoric acid were added. Finally, distilled water was added to the tube to make the final volume of 1500 μL. The tube was then vortexed to homogenize the sample and was centrifuged at 12,000×*g* for 15 min at 4 °C. Afterward, 500 μL of the supernatant was transferred in the GC vial and was analyzed using a gas chromatograph (TRACE 1300 Gas Chromatograph; Thermo Scientific, Waltham, MA, USA) coupled with a 30 m × 0.53 mm internal diameter fused silica capillary column with polar free acid phase (Stabilwax-DA, Restek Corporation, Bellefonte, PA, USA) and a flame ionization detector. The injector-port and flame ionization detector temperatures were fixed at 200 °C and 240 °C, respectively. In the temperature program, the initial temperature was held at 120 °C for 4 min after injection and then increased at 4 °C/min to 160 °C, where it was held for 4 min. Helium was used as a carrier gas. The injection volume was set at 0.5 μL, and analyses were performed. The run time for each analysis was set for 15 min. An aqueous stock standard solution was prepared with different concentrations of 0, 0.5, 1, 2, 4, 6, and 8 mmol/L with a final volume of 1500 μL. All the standard stock solutions were stored at − 20 °C until used. Data handling and processing were performed on ChromeleonTM 7.2 software (Thermo Scientific, Waltham, MA, USA).

### DNA extraction and 16S rRNA gene sequencing

Total genomic DNA was extracted from the cecal content using the QIAamp® DNA Stool Mini Kit (Qiagen, Hilden, Germany) following the manufacturer’s instruction. The concentration and integrity of bacterial DNA were determined by NanoDrop One (Thermo Fisher Scientific, Madison, WI, USA) and agarose gel electrophoresis, respectively. Amplification of the V3-V4 hypervariable regions of the 16S rRNA gene was carried out at the University of Hawaii at Manoa Advanced Studies in Genomics, Proteomics, and Bioinformatics core facility as outlined in the Illumina 16S Metagenomic Sequencing Library guideline (Illumina) with the following modification. Platinum Taq DNA Polymerase High Fidelity (Invitrogen, Life Technologies Corporation, Grand Island, NY) was used to set up the PCR reaction, Mag-Bind Total Pure NGS beads (Omega Bio-Tek) were used for PCR Clean-Ups and 35 cycles were used in PCR. Finally, amplicons were normalized, pooled, and sequenced on the Illumina MIseq sequencer.

### DNA sequence analysis

Microbial bioinformatics analysis was carried out by using CLC Genomics Workbench 12.0.1, and the CLC Microbial Genomics module. The sequencing analysis procedures were followed as described in the OTU clustering step by step tutorial (Qiagen, Hilden, Germany). Briefly, the demultiplexed sequences as fastq files were imported in the CLC workbench, which was then paired, trimmed, and filtered to remove lower coverage reads. The filtered reads were then clustered as operational taxonomical units (OTUs), based on 97% sequence similarity against the Greengenes v13_8 97% database using the CLC Microbial Genomics module. For alpha and beta diversity analysis, the phylogenetic tree was constructed using a maximum-likelihood approach based on multiple sequence alignment (MSA) of the OTUs sequences generated by MUSCLE in the workbench. The alpha diversity was estimated by calculating Simpson’s index, and Shanon entropy and was visualized by a boxplot. Beta diversity was estimated by calculating unweighted UniFrac and weighted UniFrac distances and was visualized by principal coordinate analysis (PCoA). Permutational multivariate analysis of variance (PERMANOVA) procedure was carried out to measure the significance of beta diversity. Differentially abundant taxa (order, family, and genus) were identified using one-way ANOVA on the OTU table after removing OTUs with a lower abundance of less than 10, and mean separation between the treatment groups was done by using Fisher’s Least significant difference (LSD) test in the R-studio.

### Statistical analyses

The growth performance, gene expression, VFA, and ileum histology data were analyzed using the R-studio and are presented as mean ± SEM. The mean comparisons between different treatment groups were carried out by the Tukey-Kramer test function after performing a one-way analysis of variance (ANOVA). Kruskal-Wallis pairwise test for alpha diversity and PERMANOVA test for beta diversity were used in the CLC Microbial Genomics module. The Spearman correlation analysis was carried out to determine the association between changed parameters with the differentially enriched microbial species using statistical software JMP v14 (SAS Institute Inc., Cary, NC, USA). Statistical significance was set at *P* < 0.05.

## Results

### Growth performance

**Fig. 1:**
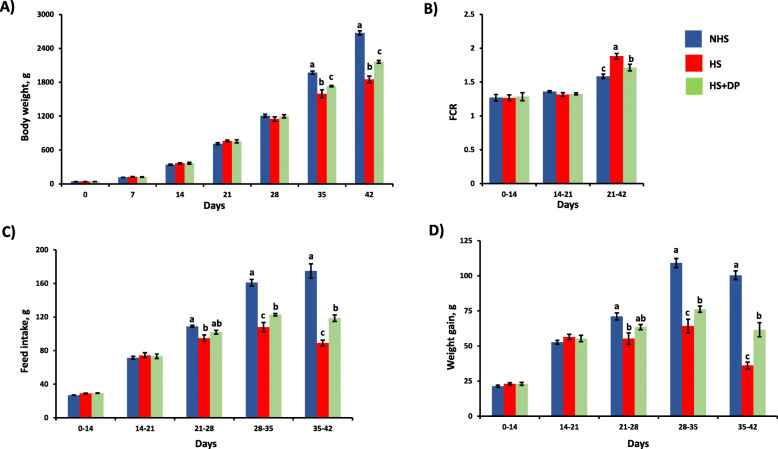
Effects of DP on the growth performance of heat-stressed broilers. **a**) Body weight, **b**) FCR, **c**) ADFI, and **d**) ADG. Data presented as the mean ± SEM. The treatment effect was statistically different at *P* < 0.05 for body weight, ADG, and ADFI; and at *P* < 0.01 for FCR. Different letters indicate the significant difference between treatment groups

There was no significant change (*P* > 0.05) in body weight between the treatment groups until 28 d (Fig. 1). However, from 35 d onwards, total body weight was significantly decreased (*P* < 0.05) in heat-stressed birds compared to the NHS group. Simultaneously, the supplementation of DP significantly increased the body weight in the heat-stressed birds as compared to the HS group. ADG was significantly decreased (*P* < 0.05) in the heat-stressed birds from 21 d as compared to the NHS group, and DP significantly improved (*P* < 0.05) the ADG from 28 d as compared to the HS group. ADFI was significantly lower (*P* < 0.05) in the heat-stressed birds from 21d as compared to the NHS, while DP significantly improved (*P* < 0.05) the ADFI from 28 d in heat-stressed birds as compared to the HS group with basal diet. During the heat stress period (21–42 d), FCR was significantly higher in the birds with normal finisher diet (HS group), while supplementation of DP significantly lowered (*P* < 0.01) FCR in the heat-stressed (HS + DP) birds.

### Effects of dried plum on the intestinal gene expression

**Fig. 2:**
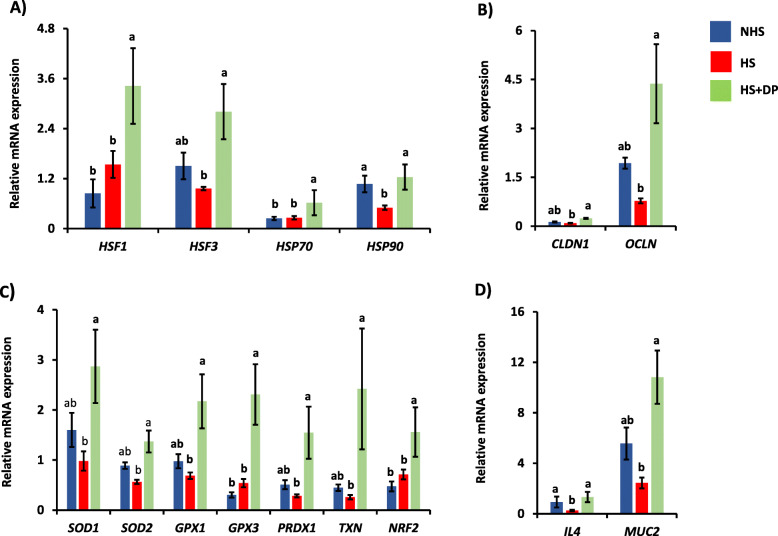
Effects of DP supplementation on the expression of **a**) heat sock protein, **b**) antioxidants related, **c**) immune-related, and **d**) tight-junction genes. Data presented as the mean ± SEM. Different letters indicate the significant difference between treatments

The expression of the heat shock protein-related genes (*HSF1*, *HSF3*, *HSP70*, and *HSP90*) across the treatment is summarized in Fig. 2a. The expression of *HSF1* and *HSF3* mRNA was significantly increased (*P* < 0.05) in the heat-stressed birds supplemented with the DP than the HS and NHS groups. The mRNA expression of the *HSP90* was significantly decreased (*P* < 0.05) in the HS group than the NHS group, whereas DP supplementation significantly increased (*P* < 0.05) the expression of *HSP90* in the heat-stressed birds as compared to the HS group. The mRNA expressions of the *HSP70* in the NHS and HS groups were similar; however, the expression was significantly increased (*P* < 0.05) in the heat-stressed birds supplemented with the DP than the NHS and HS groups.

The expression of the tight-junction-related genes (*OCLN* and *CLDN1*) across the treatment is summarized in Fig. 2b. The mRNA expression of *OCLN* and *CLDN1* was significantly increased (*P* < 0.05) HS + DP group as compared to the HS group. The expression of *OCLN* and *CLDN1* remained unchanged (*P* > 0.05) between the NHS and HS group; however, the expression of these genes was numerically lower in the HS group than the NHS group.

The expression profile of the antioxidant-related genes is presented in Fig. 2c. The dietary supplementation of DP significantly increased (*P* < 0.05) the expression of *SOD1*, *SOD2*, *GPX1*, *GPX3*, *PRDX1*, *TXN*, and *NRF2* in the heat-stressed broilers birds as compared to the HS group. In contrast, the expressions of these genes were insignificant (*P* > 0.05) between the NHS and HS groups.

The expression of immune-related genes (*IL4* and *MUC2*) across the treatment is summarized in Fig. 2d. The mRNA expression of the *IL4* was significantly decreased (*P* < 0.05) in the HS group as compared to the NHS group, while supplementation of DP significantly increased (*P* < 0.05) the expression of *IL4* in heat-stressed birds than the HS group. Supplementation of DP in the heat-stressed birds also significantly increased (*P* < 0.05) mRNA expression of the *MUC2* than the HS group. Although mRNA expression of the *MUC2* was relatively lower in the HS group than the NHS group, there was no significant difference (*P* > 0.05) in the expression of this gene in these two groups.

### Ileum histomorphology

**Fig. 3:**
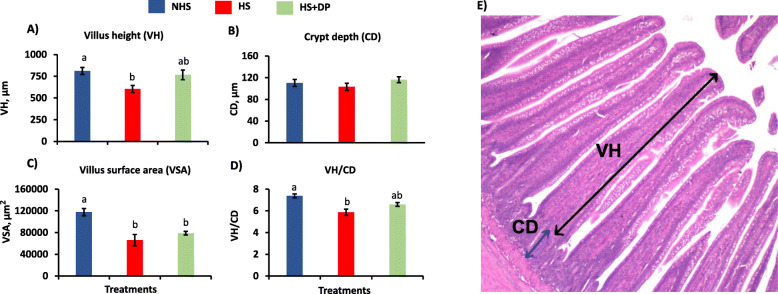
Effects of DP supplementation on the ileum histomorphology of the heat-stressed broilers. **a**) Villus height (VH), **b**) crypt depth (CD), **c**) villus surface area, **d**) villus height (VH):crypt depth (CD), and **e**) representative histological image of the ileum. The effect of treatment was statistically different at *P* < 0.05. Different letters indicate the significant difference between treatments

The villus height, and villus height to crypt depth ratio were significantly decreased (*P* < 0.05) in the HS group than the NHS group, while supplementing DP in heat-stressed birds exhibited improvement in these parameters, but statistically not significant (Fig. 3). The villus surface area was significantly decreased (*P* < 0.05) in the HS group as compared to the NHS group. Dietary supplementation of DP did not exhibit improvement for the surface area in heat-stressed birds. No significant changes (*P* > 0.05) were observed for the crypt depth.

### Volatile fatty acids (VFA)

**Fig. 4:**
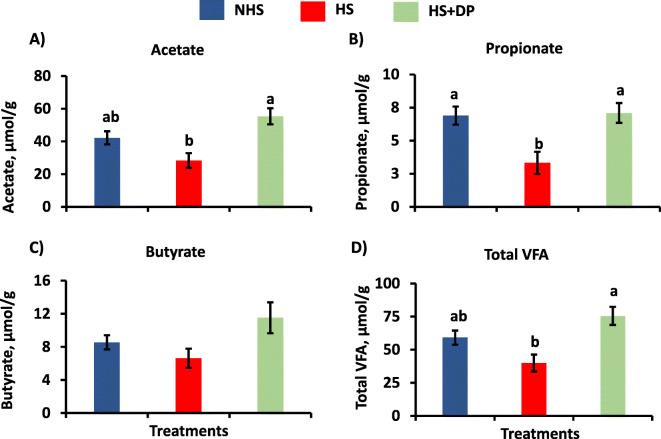
Effects of DP supplementation on the major volatile fatty acids in the cecal digesta of the heat-stressed broilers. **a**) Acetate, **b**) Propionate, **c**) Butyrate, and **d**) Total VFA. The effect of treatment was statistically different at *P* < 0.05. Different letters indicate the significant difference between treatments

The concentration of propionate was significantly decreased (*P* < 0.05) in HS groups than the NHS groups, while dietary supplementation of the DP significantly increased (*P* < 0.05) its amount in the cecal digesta as compared to the HS group (Fig. 4). Dietary supplementation of the DP also significantly increased (*P* < 0.05) the acetate concentration in the heat-stressed birds than the HS group. There was no significant difference (*P* > 0.05) in the butyrate concentration between treatment groups. Overall, the DP supplemented group (HS + DP) exhibited a significant increase in the concentration of total VFAs compared to the HS group.

### Alpha and beta diversity of cecal microbiota

**Fig. 5:**
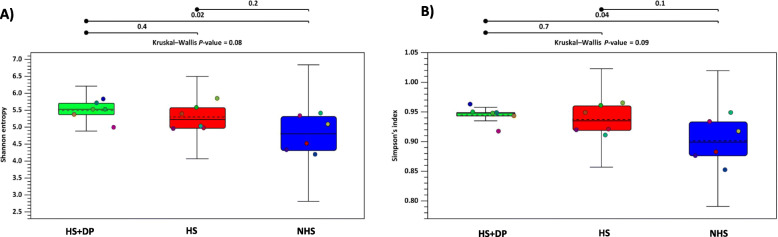
Effects of DP supplementation on microbial alpha diversity in heat-stressed birds. **a**) Shannon entropy and **b**) Simpson’s index

Alpha diversity measured the variance (diversity) within a treatment and was measured by Shannon entropy and Simpson’s index in this study (Fig. 5). Simpson’s index measures dominance or evenness while the Shannon entropy considers both the species richness and the community’s evenness. In this study, both Shannon and Simpson’s index significantly increased in the HS + DP group compared to the NHS group.

**Fig. 6:**
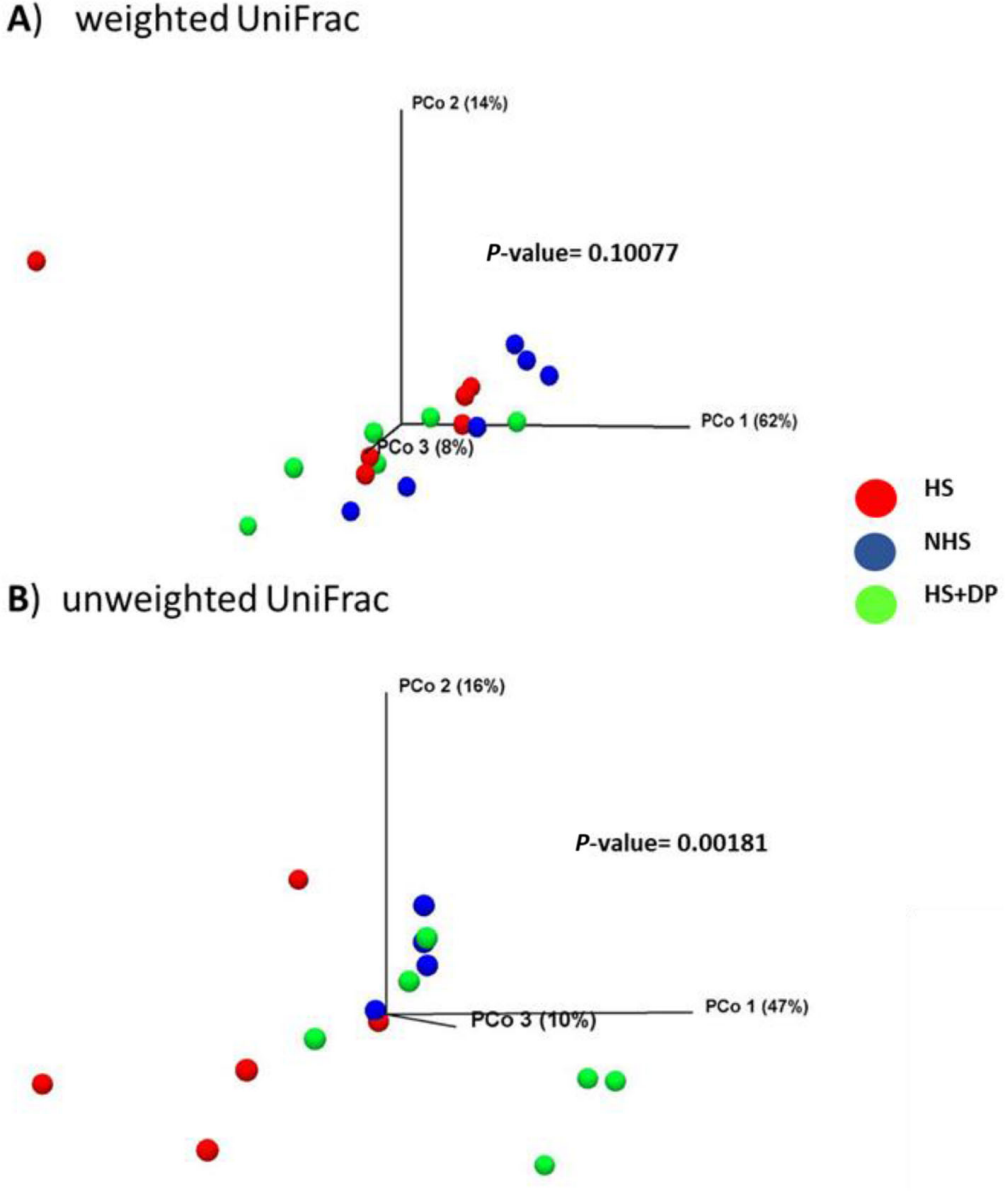
Effects of DP supplementation on microbial beta diversity in heat-stressed broilers. **a**) Weighted UniFrac and **b**) Unweighted UniFrac

Beta diversity measured the difference in the microbial composition between different environments (treatment groups) and was determined by unweighted UniFrac, and weighted UniFrac (Fig. 6). The unweighted UniFrac based PCoA reveals a significant difference in microbial composition between the treatment groups (PERMANOVA analysis, *P*-value = 0.00181).

### Cecal microbial composition

**Fig. 7:**
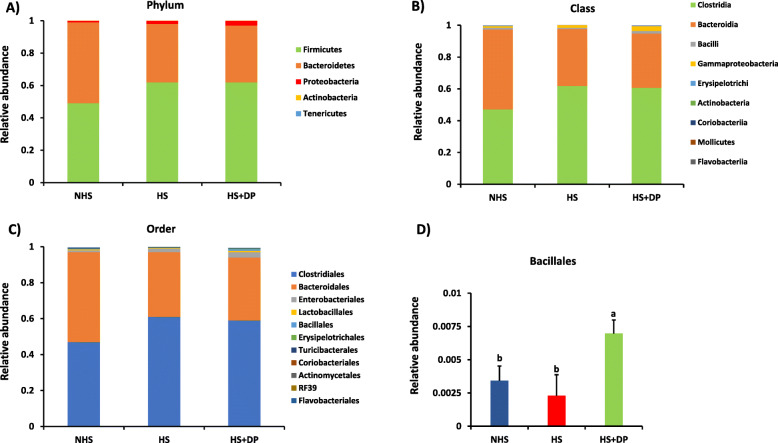
Average relative abundance of the microbiota at the phylum (**a**), class (**b**), order level (**c**), and significantly abundance microbiota at the order level (**d**)

The cecal microbiota composition at the phylum level between treatment groups after removing OTUs with low abundance is shown in Fig. 7a. Firmicutes and Bacteroidetes were the major dominant phyla across the samples in the NHS (49% and 50%, respectively), HS (62% and 36%, respectively), and HS + DP (62% and 35%, respectively).

At the class level (Fig. 7b), the cecal microbiota of the broiler birds in the NHS was dominated by the Bacteroidia (50.17%) followed by Clostridia (47.17%), while HS and HS + DP groups were dominated by Clostridia (61.83% and 60.67%), followed by Bacteroidia (35.83% and 34.167%).

At the order level (Fig. 7c), Bacteroidales was dominated in the NHS group followed by Clostridiales, whereas Clostridiales was predominant in HS and HS + DP group followed by Bacteroidales. However, the relative abundance of these dominant taxa was not significant across different groups. However, at the order level, Bacillales was significantly abundant (*P* < 0.05) in the HS + DP group compared to the HS group.

**Fig. 8:**
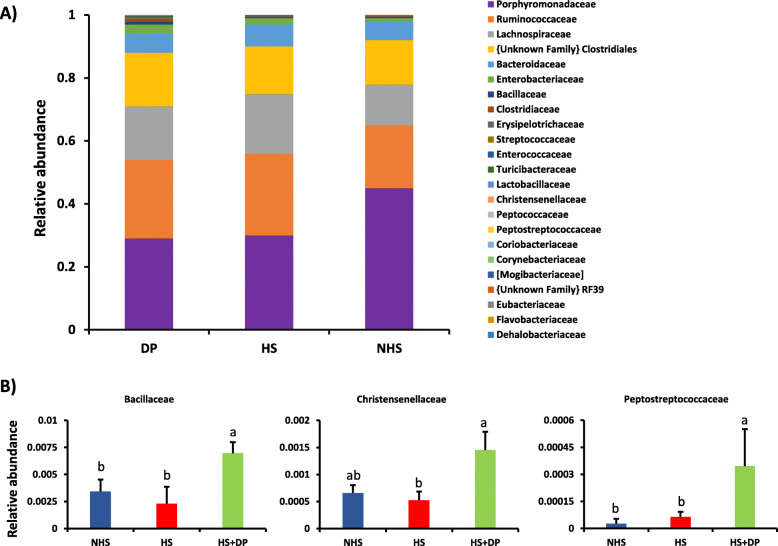
Average relative abundance of the microbiota at the family level (**a**) and significantly abundance microbiota at the genus level (**b**)

The families Porphyromonadaceae, Ruminococcaceae, and Lachnospiraceae were major dominant families in NHS, HS, and HS + DP groups (Fig. 8). However, these families were not significantly different across different groups. However, at the family level, Bacillaceae, Christensenellaceae, and Peptostreptococcaceae were significantly enriched (*P* < 0.05) in the HS + DP group as compared to the HS group.

**Fig. 9:**
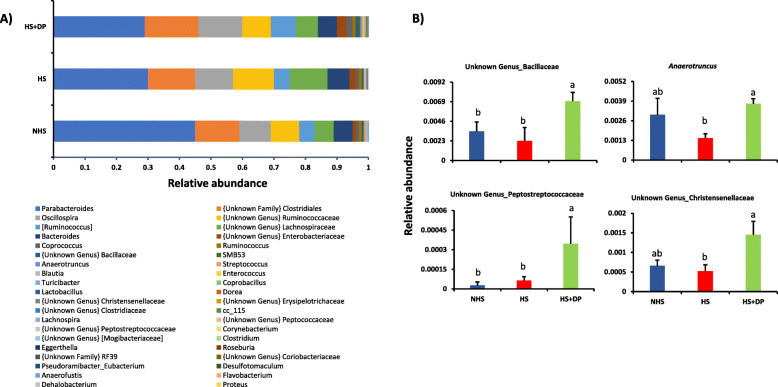
Average relative abundance of the microbiota at the genus level (**a**) and significantly abundance microbiota at the genus level (**b**)

Taxon-based analysis at the genus level revealed *Parabacteroides*, {Unknown Family} Clostridiales, and *Oscillospira* as the predominant genera (Fig. 9). The relative abundance of these genera was not statistically different across different groups. The unknown genus of Bacillaceae, *Anaerotruncus*, unknown genus of Christensenellaceae, and unknown genus of Peptostreptococcaceae were significantly enriched (*P* < 0.05) in the HS + DP group at the genus level as compared to the HS group.

### Correlation between the differential microbial species and measured parameters

Spearman’s rank correlation was calculated to identify the association between the bacterial taxa and the changed parameters considered in this study. Significant correlations observed are shown in Table 2. The antioxidant genes (*SOD2*, *TXN*, and *PRDX1*) were positively associated with order Bacillales, families Bacillaceae and Christensenellaceae*,* and genus *Anaerotruncus*. The *GPX1* was positively associated with order Bacillales, family Bacillaceae and genus *Anaerotruncus*; *GPX3* and *CLAU1* were positively associated with order Bacillales, families Bacillaceae and Peptostreptococcaceae*,* and genus *Anaerotruncus*; *NRF2* was positively associated with families Christensenellaceae and Peptostreptococcaceae; *HSF3* and *IL4* were positively associated with genus *Anaerotruncus*; and *MUC2* and *OCLN* were positively associated with order Bacillales, family Bacillaceae and genus *Anaerotruncus*. For VFA, Acetate was positively associated with the family Christensenellaceae and genus *Anaerotruncus*. While total VFAs were positively associated with order Bacillales, families Bacillaceae and Christensenellaceae, and genus *Anaerotruncus*.

**Table 2:** Spearman correlation between the differential microbial species and changed measure parameters

Variables	Differential microbial species	Spearman ρ	Prob>ρ
*SOD2*	{Unknown Genus} Bacillaceae	0.4876	0.0401*
*SOD2*	*Anaerotruncus*	0.6367	0.0045*
*SOD2*	{Unknown Genus} Christensenellaceae	0.5046	0.0327*
*SOD2*	O_Bacillales	0.4876	0.0401*
*SOD2*	F_Bacillaceae	0.4876	0.0401*
*SOD2*	F_Christensenellaceae	0.5046	0.0327*
*GPX1*	{Unknown Genus} Bacillaceae	0.4721	0.0479*
*GPX1*	*Anaerotruncus*	0.5521	0.0175*
*GPX1*	Bacillales_order	0.4721	0.0479*
*GPX1*	F_Bacillaceae	0.4721	0.0479*
*GPX3*	{Unknown Genus} Bacillaceae	0.4928	0.0377*
*GPX3*	*Anaerotruncus*	0.4716	0.0482*
*GPX3*	{Unknown Genus} Peptostreptococcaceae	0.514	0.0291*
*GPX3*	O_Bacillales	0.4928	0.0377*
*GPX3*	F_Bacillaceae	0.4928	0.0377*
*GPX3*	F_Peptostreptococcaceae	0.514	0.0291*
*PRDX1*	{Unknown Genus} Bacillaceae	0.4979	0.0355*
*PRDX1*	*Anaerotruncus*	0.5893	0.0101*
*PRDX1*	{Unknown Genus} Christensenellaceae	0.4696	0.0493*
*PRDX1*	O_Bacillales	0.4979	0.0355*
*PRDX1*	F_Bacillaceae	0.4979	0.0355*
*PRDX1*	F_Christensenellaceae	0.4696	0.0493*
*TXN*	{Unknown Genus} Bacillaceae	0.4928	0.0377*
*TXN*	*Anaerotruncus*	0.5686	0.0138*
*TXN*	{Unknown Genus} Christensenellaceae	0.5666	0.0142*
*TXN*	O_Bacillales	0.4928	0.0377*
*TXN*	F_Bacillaceae	0.4928	0.0377*
*TXN*	F_Christensenellaceae	0.5666	0.0142*
*NRF2*	{Unknown Genus} Christensenellaceae	0.4951	0.0433*
*NRF2*	{Unknown Genus} Peptostreptococcaceae	0.5029	0.0396*
*NRF2*	F_Christensenellaceae	0.4951	0.0433*
*NRF2*	F_Peptostreptococcaceae	0.5029	0.0396*
*HSF3*	*Anaerotruncus*	0.4757	0.0460*
*CLDN1*	{Unknown Genus} Bacillaceae	0.5196	0.0271*
*CLDN1*	*Anaerotruncus*	0.6739	0.0022*
*CLDN1*	{Unknown Genus} Peptostreptococcaceae	0.6004	0.0084*
*CLDN1*	O_Bacillales	0.5196	0.0271*
*CLDN1*	F_Bacillaceae	0.5196	0.0271*
*CLDN1*	F_Peptostreptococcaceae	0.6004	0.0084*
*OCLN*	{Unknown Genus} Bacillaceae	0.5134	0.0293*
*OCLN*	*Anaerotruncus*	0.7172	0.0008*
*OCLN*	O_Bacillales	0.5134	0.0293*
*OCLN*	F_Bacillaceae	0.5134	0.0293*
*MUC2*	{Unknown Genus} Bacillaceae	0.5785	0.0119*
*MUC2*	*Anaerotruncus*	0.5377	0.0214*
*MUC2*	Bacillales_order	0.5785	0.0119*
*MUC2*	F_Bacillaceae	0.5785	0.0119*
*IL4*	*Anaerotruncus*	0.4737	0.0471*
Acetate	*Anaerotruncus*	0.4964	0.0361*
Acetate	{Unknown Genus} Christensenellaceae	0.6512	0.0034*
Acetate	F_Christensenellaceae	0.6512	0.0034*
Total VFA	{Unknown Genus} Bacillaceae	0.4731	0.0474*
Total VFA	*Anaerotruncus*	0.4757	0.0460*
Total VFA	{Unknown Genus} Christensenellaceae	0.5955	0.0091*
Total VFA	O_Bacillales	0.4731	0.0474*
Total VFA	F_Bacillaceae	0.4731	0.0474*
Total VFA	F_Christensenellaceae	0.5955	0.0091*

## Discussion

Environmental heat stress negatively impacts broiler chickens’ growth performances by inducing oxidative stress and impairing the physiological parameters [2, 18, 19]. In poultry, feed intake reduces by 5% for every 1 °C rise in the temperature range of 32–38 °C [20]. In this study, heat stress significantly decreased the final body weight, ADFI, ADG, FCR, and gut health parameters. The dietary supplementing DP (2.5%), on the other hand, improved the growth performance (body weight, ADG, ADFI, and FCR) along with the expression of heat shock protein-related, antioxidant related, immune-related, and tight-junction related genes in the heat-stressed birds. Besides, DP supplementation also improved intestinal integrity, increased butyrate along with total VFAs concentration in the cecum, and enriched the relative abundance of beneficial bacteria in the cecum. Improvement in the production performance while fortifying the diet with 2.5% DP can be attributed to antioxidants, polyphenols, vitamins, and minerals. Similarly, FCR in heat-stressed birds supplemented with DP was improved, which can be ascribed to a better weight gain and feed utilization.

The gastrointestinal tract (GIT) is considered as the main target of heat stress [21]. To delineate the potential mechanism by which DP improved the growth performances of heat-stressed birds, different gut health parameters were analyzed. The ileum, the terminal part of the small intestine, is associated with the absorption of most of the nutrients in the poultry and is more prone to heat stress [22]. In response to the elevated temperature, the cell possesses two major kinds of protective mechanisms to maintain normal cellular functions: firstly, by the production of the HSPs, and secondly, by increasing the production of the antioxidants inside the cell. The HSPs are a group of proteins that are produced by the cell under stress conditions. These proteins are transcriptionally regulated by heat shock factors (HSFs) [23]. Both HSP70 and HSP90 acts as chaperons, ensure proper folding of the proteins, and have cytoprotective action [24]. Besides this, an increased expression of *HSP70* was increased in broilers digestive enzyme activity [25] and promote the production of glutathione (GSH), superoxide dismutase (SOD), and total antioxidant capacity (TAOC) [26]. Previous studies have reported that *HSFs* and *HSPs* are up-regulated during acute heat stress [22, 27]. In this study, however, the chickens subjected to heat stress had significantly lower expression of *HSF3* and *HSP90*. The expressions of HSFs and HSPs are found to be tissue-specific and varied with the stress duration (i.e., acute vs. chronic stress) [28]. Moreover, the biphasic expression pattern of HSP70s was observed in the cattle [29]. Therefore, spatiotemporal expressions of these HSFs and HSPs are required to understand the functionality of these molecules. Interestingly, the expression of *HSF1*, *HSF3*, *HSF70*, and *HSP90* were increased in heat-stressed birds supplemented with DP. The increased expression of *HSP70* and *HSP90* is likely due to the increased expression of *HSF1* and *HSF3.* The role of HSF1 in the induction of HSP70 [30], and HSF3 in promoting the expression of all HSPs [31] in the chicken are widely known. The correlation analysis showed that the expression of *HSF3* was positively associated with cecal bacteria *Anaerotruncus*. This relationship warrants further investigation.

In response to the oxidative stress elicited by heat stress in poultry, Nrf2—a redox-sensitive nuclear transcriptional factor—is translocated in the nucleus, where it binds in the promotor region of the antioxidant response element in the DNA, leading to the production of different antioxidants [32]. Therefore, to better understand the antioxidant status of the heat-stressed birds, different antioxidant related genes such as *SOD1*, *SOD2*, *GPX1*, *GPX3*, *PRDX1*, *TXN*, and *NRF2* were analyzed. SOD1 and SOD2 are isoforms of superoxide dismutase (SOD). SOD1, cytosolic Cu/ZnSOD, is mainly localized in the cytoplasm, mitochondrial intermembrane spaces, nucleus, lysosomes, and peroxisomes, whereas SOD2 is mitochondrial manganese (Mn) containing enzymes [33]. Superoxide radical is the predominant free radicals produced inside the cell [34]. SODs catalyze the superoxide radical into hydrogen peroxide; thus, it is considered the main element of the first level of antioxidant defense in the cells [35]. Similarly, GPX1 and GPX3 are the selenium-dependent forms of glutathione in avian species. GPX1 is mainly localized in the cytoplasm and mitochondria, while GPX3 is most abundant in the plasma. GPXs catalyzes the reduction of hydroperoxides and H_2_O_2_ by glutathione [36]. PRDX1 is a member of the Peroxiredoxins family, which uses thioredoxin to reduce H_2_O_2_, hydroperoxides, and proximities to balance ROS inside the cell [37]. TXN is a key member of the thioredoxin system-important antioxidant system in defense against oxidative stress in the cell. Moreover, thioredoxin is also involved in immune response, DNA and protein repairs, and cell death [38]. In this study, the expression of *NRF2*, *SOD1*, *SOD2*, *GPX1*, *GPX3*, *TXN*, and *PRDXN* were significantly improved in the heat-stressed birds supplemented with the DP. Indeed, significant expression of the *NRF2* along with antioxidants (*SOD1*, *SOD2*, *GPX1*, and *GPX3*) indicate that DP was able to activate Nrf2 mediated antioxidants enzymes in the heat-stressed birds. Previous experiments on phytochemicals have demonstrated a vital role of polyphenols in the activation of the Nrf2 mediated antioxidants enzymes [6, 39]. In agreement with those studies, polyphenols present in the DP possibly may have played a similar role resulting in the upregulation of antioxidant genes in heat-stressed birds, thus reducing ROS and lipid peroxidation within the cell.

The intestinal epithelial tight junctional barrier plays a vital role against paracellular penetration of the pathogenic bacteria, endotoxins, and feeds associated antigens [40–42]. Occludin (OCLN) and claudin (CLDN) are the major transmembrane proteins that make up the tight junctions. Occludin helps regulate paracellular permeability and plays a key role in cellular structure and barrier function. Claudins form the backbone of tight junctions and play a significant role in the tight junction ability to seal the paracellular space [43]. During heat stress, peripheral circulation of the blood increased as a result of blood flow in the epithelium in the intestine is reduced, resulting in hypoxia, which leads to the disruption of tight junction, reduced intestinal integrity, and increases intestinal permeability that is found to increase the circulating endotoxins [44]. In this study, the expression of the *OCLN* and *CLDN1* was significantly increased in the ileum of heat-stressed birds supplemented with DP. This result indicates that dietary DP improved the intestinal tight-junction related genes in the heat-stressed birds, which may be attributed as one of the reasons for the improvement in the production performance in heat-stressed birds. Flavonoids, polyphenol compounds, exhibit promotive and protective effects on intestinal tight junction barrier functions [45]. Improved expression of the tight junction in the heat stress in the DP supplemented birds may be due to its flavonoids. Besides, Spearman correlation revealed the positive association of *OCLN* with Bacillales, Bacillaceae and *Anaerotruncus*; and *CLDN1* with Bacillales, Bacillaceae, Peptostreptococcaceae, and *Anaerotruncus*.

To delineate the effects of DP on the intestinal immune system, the different immune-related genes in the ileum broilers were analyzed. The expression of *IL4* and *MUC2* was significantly increased by supplementing DP. IL4 is a cytokine that plays a vital role in regulating the immune system and cellular homeostasis [46]. Spearman correlation analysis revealed a positive association of *IL4* with *Anaerotruncus*. Thus, a significant expression of *IL4* indicates the enhanced immune response in heat-stressed birds. Mucin is the major constituent of mucus layers and plays a pivotal role in protecting the gut from pathogens, acidic chyme, and digestive enzymes, influencing nutrient absorption and digestion [47]. The significant expression of *MUC2* has probably played a protective role against pathogens along with enhancing nutrient digestion in heat-stressed broilers.

High temperature has been shown to impair the intestinal integrity in the broilers [48, 49]. Consistent with those findings, the result of this study demonstrated that the morphology of the ileum was damaged with heat stress, which could be due to the epithelial shedding in response to intestinal ischemia [50]. Although statistically not significant, supplementing DP improved the villus height and villus height to crypt depth ratio in heat-stressed birds. VFAs are found to exert tropic effects on intestinal morphology [51]. In this regard, the beneficial effect of DP on the intestinal morphology may be exhibited due to increased colonization of the beneficial bacteria along with the production of higher amounts of VFAs in heat-stressed birds.

Acetate, propionate, and butyrate are the major VFA produced by the fermentation of the dietary fibers in the poultry’s cecum. VFAs are found to play a vital role in the gut and immune homeostasis. Acetate, dominant VFA, is involved in the muscles’ glycolytic pathway; propionate is associated with gluconeogenesis in the liver, while the butyrate serves as a fuel for colonocytes [52]. In this study, the amount of acetate, propionate, and total VFA was increased significantly in the heat-stressed birds supplemented with the DP, while the amount of the butyrate was found to increase numerically. There was a positive association of acetate with Christensenellaceae and *Anaerotruncus*; and a positive association of total VFAs with Bacillales, Bacillaceae, Christensenellaceae, and *Anaerotruncus*. These improvements in the VFAs can be attributed to the higher level of dietary fibers present in the DP along with the enriched bacteria. Moreover, these results of VFA help corroborate our finding of improved growth performance, ileum histomorphology, and expression of tight-junction genes.

Gut microbiota plays a crucial role in the intestinal health and growth performances of birds. On the other hand, high temperature has been associated with the dysbiosis of the cecal microbiota in poultry. Supplementation of DP significantly improved bacterial richness and diversity of cecal microbiota in heat-stressed birds, which may be attributed due to the high dietary fibers and polyphenols in the DP. The results of this study show that heat stress in broilers exhibited a significant change in unweighted UniFrac measures of beta diversity, which was consistent with the previous study [53] and indicate that bacteria having relatively lower abundance were significantly different between the treatments.

Afterward, significantly abundant taxa were determined. At the order level—Bacaillale, at the family level—Bacillaceae, Christensenellaceae, Peptostreptococcaceae, and at the genus level - unknown genus of Bacillaceae, *Anaerotruncus*, unknown genus of Christensenellaceae, and unknown genus of Peptostreptococcaceae were significantly enriched in the heat-stressed birds supplement with the DP*.* A positive correlation was observed between Bacillaceae and different observed parameters (*TXN*, *PRDX1*, *CLDN1*, *OCLN*, *MUC2*, and total VFAs). In drosophila larvae, the relative abundance of the Bacillaceae was significantly lower in cancerous larvae than those without the tumor. Furthermore, they hypothesized Bacillaecae could eliminate cancer cells at the beginning of carcinogenesis potentially by stimulating the immune system [54]. Additionally, *Bacillus* spp. member of Bacillaceae is found to display antimicrobial, antioxidant and immune-modulatory activity in the host and has gained significant attention in the past decade as a potential probiotic [55]. Considering these facts, along with our observed results, we can conclude that significant enrichment of Bacillaceae in heat-stressed broiler birds has played a vital role in improving the immune status by enhancing different antioxidants, tight-junction genes, and VFAs.

The family Christensenellaceae belongs to the phylum Firmicutes and plays a vital role in human health [56]. The positive association of Christensenellaceae with antioxidant-related genes (*TXN* and *NRF2*), acetate, and total VFAs was observed in our study. Christensenellaceae is considered one of the signature taxa of a healthy gut and are depleted in conditions associated with inflammation [57]. Moreover, the significant abundance of the Christensenellaceae was found to have a negative correlation with the visceral fat mass [58] and is also negatively associated with the total cholesterol and low-density cholesterol (bad cholesterol). Interestingly, a higher amount of abdominal fat is found in heat-stressed poultry [59]. Taken together, our study showed a significant abundance of the Christensenellaceae in the DP, demonstrates its beneficial effect on the gut. Christensenellaceae are associated with diets higher in fibers [56]. Thus, we can speculate that the significant abundance of the Christensenellaceae in heat-stressed broiler birds supplemented with DP is due to its higher dietary fiber content.

The family Peptostreptococcaceae belongs to phylum Firmicutes and is found as the normal commensal of the gut. They are reported to be higher in the gut of healthy rats than one with dysbiosis [60]. This highlights its role in maintaining gut homeostasis. Studies have shown that Peptostreptococcaceae are involved in the production of VFAs from amino acids. Thus, the significant abundance of the Peptostreptococcaceae may also have involved in increasing the production of VFAs in the ileum of heat-stressed birds supplemented with DP [61].

Finally, at the genus level, *Anaerotruncus* was a significant enrichment in heat-stressed birds supplemented with DP. The *Anaerotruncus* was positively associated with *IL4*, *HSF3*, *CLAU1*, *OCLN*, *MUC2*, acetate, and total VFA in our study. Interestingly, *Anaerotruncus* is among the 17 strains of the intestinal bacteria found to stimulate the regulatory T-cells and attenuate the inflammation in a mouse colitis model [62]. Also, antibiotic-induced noninfectious colitis in humans is reported to be treated by the community of 17 intestinal bacteria that include *Anaerotruncus* by fecal transplantation [63]. Moreover, probiotic supplementation during the recovery phase after antibiotic administration was found to suppress the growth of *Shigella* and *Escherichia*, while blooming *Anaerotruncus *spp. [64]. Considering these, the significant abundance of *Anaerotruncus* in our study highlights its potential role in attenuating inflammation and enhancing immunity in the intestine.

There have been several studies highlighting the role of vitamins (Vit A, E, and C) and minerals (Mn, Se, Zn, Mg, and Cu) in mitigating heat stress in poultry [18]. DP is a mixture of bioactive compounds - vitamins, minerals, and polyphenols [9]. A simple calculation shows that the supplementation provides 0.15 mg Vit C, 0.108 mg Vit E, 0.975 μg Vit A, 0.0.0748 mg Mn, 0.075 μg Se, 0.11 mg Zn, and 10.25 mg Mg per kg of the experimental diet fed to the chicken. Then on top levels provided to the premix, DP is the likely reason for the DP’s beneficial effects in the heat-stressed broiler birds. Moreover, higher amounts of glucose obtained from the DP diet also attributed the beneficial effects in the heat-stressed broiler birds. Further nutrient profiling of DP will help precise feed formulation and evaluate its additional performance and health benefits in poultry.

## Conclusion

The results of this study revealed that the dietary supplementation of DP significantly improved the body weight, ADG, ADFI, FCR, ileum histomorphology, cecal VFA production, and expression of heat sock, antioxidant, immune, and tight junction related genes in the heat-stressed birds. Moreover, DP was also able to improve the relative abundance of beneficial bacteria in the chicken gut. Thus, considering the benefits of supplementing DP in heat-stressed birds, the dietary supplementation of DP can be considered a potential strategy to mitigate the negative effects of heat stress in poultry.

## Supplementary Information

**Additional file 1: Table S1.** Nutrient composition of dried plum. **Table S2.** Primers used to 838 quantify the expression of the genes by qPCR. **Fig. S1.** Experimental design.

## Data Availability

The sequence data reported in this paper have been deposited in the NCBI database (Metagenomic sequencing data: PRJNA688117).

## Abbreviations

ADFI: Average daily feed intake
ADG: Average daily gain
ALA: Alpha-lipoic acid
ANOVA: Analysis of variance
CD: Crypt depth
cDNA: Complementary deoxyribonucleic acid
CLDN1: Claudin 1
DP: Dried plum
FCR: Feed conversion ratio
GC: Gas chromatography
GPX1: Glutathione Peroxidase 1
GPX3: Glutathione Peroxidase 3
HS: Heat stress
HSF1: Heat shock factor 1
HSF3: Heat shock factor 3
HSP70: Heat Shock Protein 70
HSP90: Heat Shock Protein 90
IL4: Interleukin 4
MUC2: Mucin 2
OCLN: Occludin
OTU: Operational taxonomic unit
PCR: Polymerase chain reaction
PRDX1: Peroxiredoxin 1
qPCR: Quantitative polymerase chain reaction
ROS: Reactive oxygen species
SOD1: Superoxide dismutase 1
SOD2: Superoxide dismutase 2
TMA: Trimethyl acetate
TXN: Thioredoxin
VFA: Volatile fatty acid
VH: Villus height
