# Supplementary material for: Dietary supplementation of dried plum: a novel strategy to mitigate heat stress in broiler chickens
Source: J Anim Sci Biotechnol. 2021 Mar 30;12:58. doi: 10.1186/s40104-021-00571-5 (PMC8008564; doi:10.1186/s40104-021-00571-5)
Supplement: Supplementary file 1 — Additional file 1: Table S1. Nutrient composition of dried plum. Table S2. Primers used to 838 quantify the expression of the genes by qPCR. Fig. S1. Experimental design. [file 40104_2021_571_MOESM1_ESM.docx]

**Table S1** Nutrient composition of dried plum.

| Items | Dried plums |
| --- | --- |
| Serving, g | 100 |
| Energy, kcal | 240 |
| Total carbohydrate, g | 63.88 |
| Total sugars, g | 38.13 |
| Glucose, g | 25.46 |
| Fructose, g | 12.45 |
| Sucrose, g | 0.15 |
| Starch | 5.11 |
| Total dietary fiber, g | 7.1 |
| Sorbitol, g | 12 |
| Protein, g | 2.18 |
| Fat, g | 0.38 |
| Moisture, g | 30.92 |
| Ca, mg | 43 |
| K, mg | 732 |
| Fe, mg | 0.93 |
| Mg, mg | 41 |
| P, mg | 69 |
| Cu, mg | 0.281 |
| Mn, mg | 0.299 |
| Se, µg | 0.3 |
| Zn, mg | 0.44 |
| Vitamin A, µg RAE | 39 |
| Beta-carotene, µg | 394 |
| Alpha-carotene, µg | 57 |
| Beta-cryptoxanthin, µg | 93 |
| Lutein + zeaxanthin, µg | 148 |
| Vitamin C, mg | 0.6 |
| Vitamin E, mg (α-tocopherol) | 0.43 |
| Vitamin K_1_, µg | 59.5 |
| Thiamin (B_1_), mg | 0.051 |
| Riboflavin (B_2_), mg | 0.186 |
| Niacin, mg | 1.882 |
| Panthotenic acid, mg | 0.422 |
| Vitamin B_6_, mg | 0.205 |
| Folate, µg | 4 |
| Choline, mg | 10.1 |

Data from Stacewicz-Sapuntzakis [9].

**Table S2** Primers used to quantify the expression of the genes by qPCR.

| **Gene** | **Accession No.** | **Primer sequence*** | **Amplicon, bp** |
| --- | --- | --- | --- |
| SOD1 | NM_205064.1 | F: CAACACAAATGGGTGTACCA | 119 |
|  |  | R: CTCCCTTTGCAGTCACATTG |  |
| SOD2 | NM_204211.1 | F: CCTTCGCAAACTTCAAGGAG | 160 |
|  |  | R: AGCAATGGAATGAGACCTGT |  |
| GPX1 | NM_001277853.2 | F: AATTCGGGCACCAGGAGAA | 101 |
|  |  | R: CTCGAACATGGTGAAGTTGG |  |
| GPX3 | NM_001163232.2 | F: GAGGGAGAAGGTGAAATGCT | 192 |
|  |  | R: CCCAGCTCATTTTGTAGTGC |  |
| TXN | NM_205453.1 | F: GGCAATCTGGCTGATTTTGA | 79 |
|  |  | R: ACCATGTGGCAGAGAAATCA |  |
| PRDX1 | NM_001271932.1 | F: GGTATTGCATACAGGGGTCT | 101 |
|  |  | R: AGGGTCTCATCAACAGAACG |  |
| NRF2 | NM_205117.1 | F: CCCTGCCCTTAGAGATTAGAC | 248 |
|  |  | R:CAAGTTCATGTCCTTTTCTCTGC |  |
| HSF1 | NM_001305256.1 | F: AAGGAGGTGCTCCCAAAGTA | 221 |
|  |  | R: TTCTTTATGCTGGACACGCTG |  |
| HSF3 | NM_001305041.1 | F: TTCAGCGATGTGTTTAACCCT | 244 |
|  |  | R: GGAGGTCTTTTGGATCCTCT |  |
| HSP90 | NM_001109785.1 | F: GATAACGGTGAACCTTTGGG | 120 |
|  |  | R: GGGTAGCCAATGAACTGAGA |  |
| HSP70 | NM­_001006685.1 | F: TCTCATCAAGCGTAACACCAC | 104 |
|  |  | R: TCTCACCTTCATACACCTGGAC |  |
| OCLN | NM_205128 | F: CCGAGGACAGCCCTCAATAC | 82 |
|  |  | R: CTTTGGTAGTCTGGGCTCCG |  |
| CLDN1 | NM_001013611 | F: TACCCCAAAAATGCCCCCTC | 109 |
|  |  | R: GCGGCATTGTAGTGTCCTCT |  |
| MUC2 | NM_001318434 | F: GTGGTCTGTGTGGCAACTT | 71 |
|  |  | R: GTCTCTTGCAGCCCATTCCT |  |
| IL4 | NM_001030693 | F: TGTGCCCACGCTGTGCTTACA | 155 |
|  |  | R: CTTGTGGCAGTGCTGGCTCTCC |  |

*F=Forward; R= Reverse


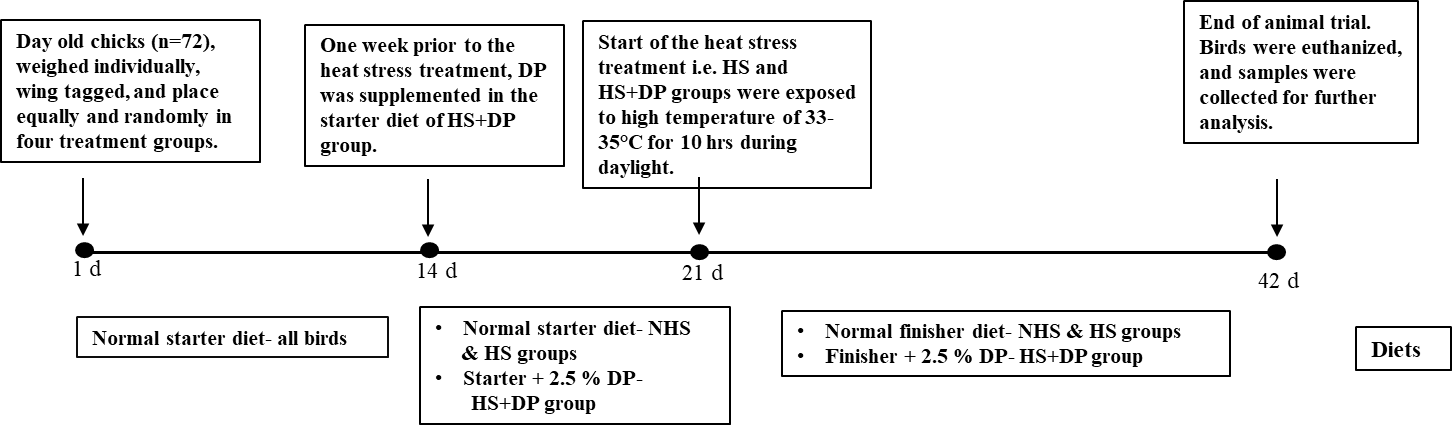


**Fig. S1** Experimental design.
